# Temporally Robust Eye Movements through Task Priming and Self-referential Stimuli

**DOI:** 10.1038/s41598-017-07641-7

**Published:** 2017-08-03

**Authors:** Eun-Soo Jung, Dong-Gun Lee, Kyeongho Lee, Soo-Young Lee

**Affiliations:** 1 School of Electrical Engineering, Korea Advanced Institute of Science and Technology, Daejeon, South Korea; 2 Brain Science Research Center, Korea Advanced Institute of Science and Technology, Daejeon, South Korea

## Abstract

Studies have demonstrated connections between eye movements and attention shifts. However, little is known about the general factors that contribute to the self-consistency of idiosyncratic scanpaths as a function of attention shifts over time. The present work repeatedly measured human eye movements at various time intervals that ranged from less than one hour to one year between recording sessions. With and without task context, subjects observed multiple images with multiple areas of interest, including their own sporadically interspersed facial images. As reactions to visual stimuli, the eye movements of individuals were compared within and between subjects. We compared scanpaths with dynamic time warping and identified subjects based on the comparisons. The results indicate that within-subject eye movement comparisons remain more similar than between-subject eye movement comparisons over time and that task context and self-referential stimuli contribute to the consistency of idiosyncrasies in attention shift patterns.

## Introduction

Eye movements provide information about individuals as they respond to external stimuli. Perceptual attention affects human eye movements^1, 2^, and unconscious shifts of attention also direct involuntary eye movements^3, 4^. Different goals and visual stimuli induce different viewing patterns^5, 6^. Even the same visual stimulus attracts attention differently between individuals as each individual has distinctive preferences^7^, memories^4, 8^, or cravings^9–11^. According to the scanpath theory, an individual tends to scan visual stimuli in unique fixation sequences^12, 13^. These studies are distinct from research on bottom-up visual saliency, which focuses on common fixation locations over observers^14^, and ground research into idiosyncratic eye movements.

Studies on eye movements support their potential applications, such as biometric security systems^15, 16^, marketing research^17^, and monitoring of subject physical states^18^. However, established studies on idiosyncratic scanpaths due to attention shifts across multiple objects have been limited, particularly on their temporal robustness. Research has shown that images with many areas of interest (AOIs) can increase between-observer eye movement differences more than images with relatively fewer AOIs^19^. Thus, we expected that multiple unrelated images presented together on a single screen would induce variations in subjects’ eye movements more than each image individually. One study^20^ observed individuals’ distinctive eye movement features during comparison of two simultaneously presented facial patterns. However, that study focused on specific eye movements involved in face perception with a small data set. In this study, we observed scanpath idiosyncrasies over time and investigated the influence of two factors on their temporal consistency. The first factor involves attention shifts within the context of a task, and the second involves the contents of visual stimuli.

Desires or preferences can drive individuals’ spontaneous attention^7, 9–11^, and these factors do not strongly correlate with bottom-up saliency factors^21^. To enhance this top-down modulation of eye movements, we assigned a preference selection task to participants during an experiment. Research on eye tracking also has demonstrated that gaze bias towards a chosen item or face is larger during preference selection than rounder face selection^22^ or dislike selection tasks^23^. Therefore, we expected that subjects’ preferences would best reflect unique personal experiences and would induce more idiosyncratic scanpaths; we also hypothesized that a task itself would enhance subjects’ interests and motivation.

The second factor regards the ability of visual stimuli to elicit scanpaths. Thus far, the relationship between the temporal robustness of individual scanpaths and the contents of visual stimuli has not been adequately explored. Whereas most previous research limited the category of visual stimuli^15, 20–24^, we did not restrict stimulus contents to draw diverse memories and preferences from subjects. We expected that particular visual stimuli would evoke self-referential memories, thus drawing stronger attraction or preference from each subject and helping to build more temporally robust scanpaths. Particularly, pictures of each subject’s own face were collected and presented in our experiment. Research has demonstrated that a subject’s own name or face distracts attention from unrelated task performance, although this effect diminishes with repeated exposure^25, 26^. Likewise, eye movements have been shown to be less idiosyncratic during observation of unfamiliar faces than during observation of more recognizable, familiar faces^27, 28^. As one’s own face is a “familiar” face for each individual, we hypothesized that self-face images would attract spontaneous attention at least as long as few stimuli are used to prevent attention wear out.

To investigate idiosyncratic scanpaths, we repeatedly conducted an experiment in which sets of images were presented to subjects. Eye movements of subjects were recorded as attention shifts within and between these images. These eye movements were observed over a period of two weeks (and additionally over a year), their idiosyncrasies were confirmed, and the influence of the suggested two factors on their temporal consistency was assessed with various methods.

## Materials and Methods

This section describes the process of data acquisition and methods of scanpath comparisons. Eye movements were recorded with and without a preference selection task, and the raw gaze data were processed into continuous scanpaths. Pairwise comparisons were performed on scanpaths of each stimulus, and recording time intervals between scanpath pairs were defined. More information regarding the scanpath comparisons can be found in the Supplementary Text.

### Subjects

The purpose and every process in the experiment were approved by the KAIST Institutional Review Board. All methods were performed in accordance with the relevant guidelines and regulations, and written informed consent was obtained from every subject. Twenty-seven subjects (fifteen males and twelve females), who do not need glasses or feel comfortable with contact lenses and do not have any history of mental disorders, were selected for the experiment from a pool of university student applicants. The ages of those selected ranged from 20 to 30 with an average age of 23.2 years (*s*.*d*. = 3.07). The subjects were electronically paid after each participation in the experiment.

Since the purpose of this research is observing the idiosyncrasies of individuals and their temporal consistencies, each subject had to repeatedly participate in the experiment on separate days. We prespecified a sample size of at least 25 participants with at least three repetitions. These determinations were made on the basis of time, resources, and labour constraints, without the use of power analysis.

### Visual stimuli

Images of various categories were collected from the Internet, Facial Recognition Technology database^29^, ImageNet database^30^, and photographs that an author took. Additionally, three different self-images were collected from each subject and added to the library. In total, 440 images were gathered for the experiment (and divided into rough categories in Supplementary Table S1). The unique focal object or face in every selected image was larger than 250 × 250 pixels. All images were cropped around focal objects or faces and resized to 350 × 350 pixels. Colours were removed, and illumination was controlled to reduce the saliency effect, which can attract subjects’ bottom-up attention.

**Figure 1:**
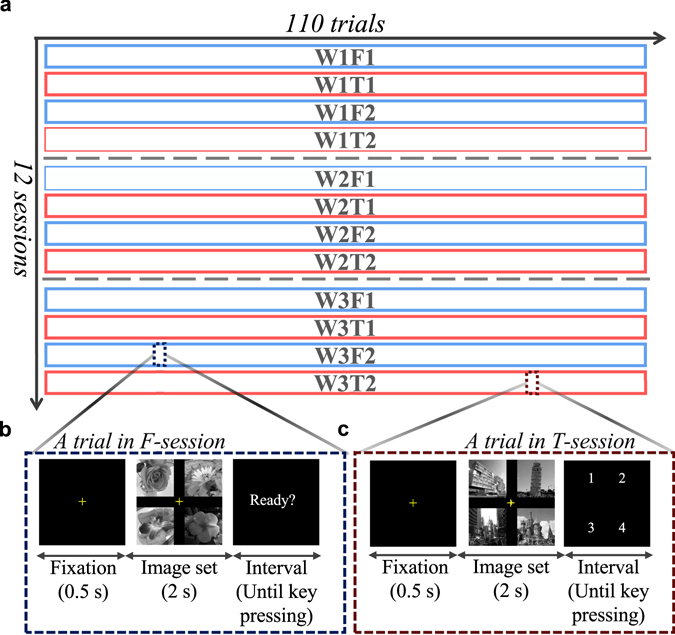
Overall procedure of the experiment. (**a**) Organization of Free and Task sessions for each subject. Sessions were named according to the week of recording and the type of session. The same 110 image sets were presented in a different order in each session. (**b**) A trial in a Free session. An image set was presented in a trial. A subject observed each image set with no task in Free sessions. (**c**) A trial in a Task session. After observing an image set, a subject had to choose their favourite image.

Sets of four different images were randomly assembled, with each constituting one image set. However, three self-images from the same subject were included in three different image sets. Each set of images was arranged on an 800 × 800-pixel black background with one image at each corner, and a yellow fixation cross was placed at the centre. From the library of 440 images, 110 unique image sets were created (two example image sets are shown in Fig. 1). Image sets were presented on a 23-inch (~59 cm diagonal) monitor with pixel resolution set at 1680 × 1050. The width (23 cm) and length (23 cm) of each image set subtended approximately 22° × 22° of the visual angle for a subject sitting approximately 60 cm away from the monitor. Similar experimental settings are commonly used in studies on eye movements^31^. The pixel area occupied by each different image within an image set was defined as a separate AOI. As the effective human visual angle is typically up to 30°^32^, subjects were expected to be simultaneously visually aware of all four AOIs of an image set at the start of a trial, and their eye gaze was predicted to spontaneously move from the fixation point to anywhere in this visual field during the exposure period (2 s).

### Apparatus

A Tobii × 120 table-mounted eye tracker (Tobii Technology AB, Sweden) with an accuracy of 0.5° and a sampling rate of 120 Hz was used for the experiment^33^. Every session started after calibration to attune the equipment to each subject’s ocular characteristics and accurately calculate the gaze positions. The calibration was repeated until the calibration error was less than 2°.

### Experimental procedure

For each subject, the experiment was repeated on three experimental days with a one-week interval between each day. Four sessions were recorded each day with a short break (about five minutes) between the second and third session. In total, there were twelve sessions of recordings over three weeks for each subject. There were two types of sessions: “Free (viewing) sessions”, where the subjects were asked to observe the stimuli freely, and “Task sessions,” where the subjects had to choose a favourite image from each image set. Free sessions and Task sessions were administered on an alternating basis. Sessions were named according to when they were recorded and by the type of session (e.g., the first Free session recorded in the third week is W3F1 for each subject) (Fig. 1a).

Each session consisted of 110 trials with each presenting a different image set. Namely, every image was presented only once in a session. In each trial, a fixation cross was displayed at the centre of the screen for 0.5 s, followed by an image set for 2 s. Then, a sign with “Ready?” appeared on the screen for Free sessions, or a sign with numbers from 1 to 4 appeared for Task sessions (Fig. 1b and c). At the “Ready?” signal, the subjects had to press a button to move to the next stimulus. For Task sessions, subjects had to choose the number corresponding to their favourite image in the image set. The duration of each session varied depending on each subject; however, most of the subjects completed a session in 10 minutes. A platform offered by Tobii Studio^TM^ (Tobii Technology, Sweden) was used to control the experimental procedure and collect subjects’ eye movements.

The same 110 image sets were differently ordered for each session (we changed the orders with the random permutation function in MATLAB R2014a (MathWorks Inc., USA)), but the subjects were not informed of this fact. All subjects viewed the same orders during the same sessions. Three dummy trials were added to the sessions in the second and third weeks to make the subjects think that not all of the image sets were repeated. Image sets in the dummy trials were produced in the same way as other image sets and were composed of images that were not included in any other sets. Eye movements during the dummy trials were discarded and were not used for further analyses.

### Data preprocessing

**Figure 2:**
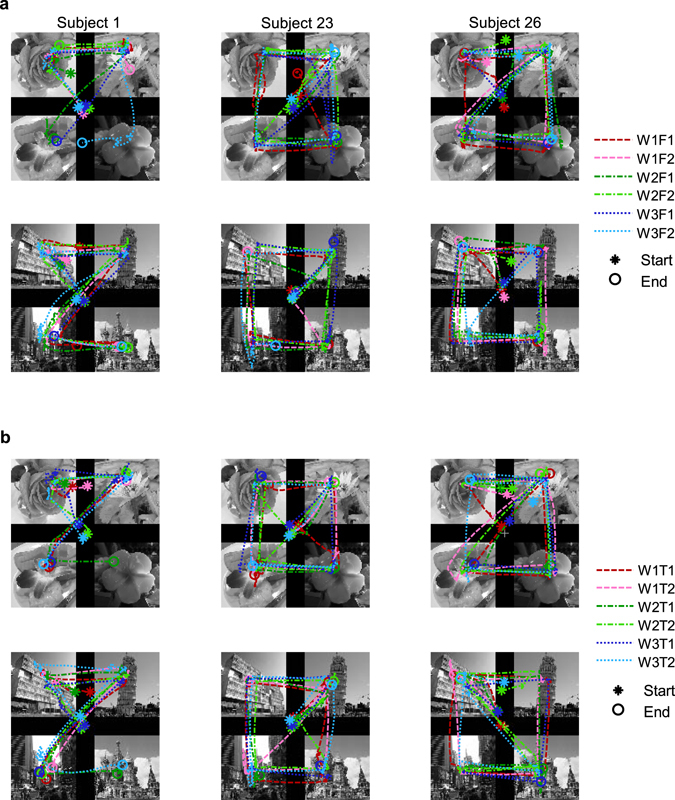
Scanpath examples. Examples of scanpaths in a 2D coordinate system for (**a**) Free sessions and (**b**) for Task sessions. The starting point of each scanpath is marked with an asterisk (*) and the end point with a circle (o). Each subject’s scanpaths during observations of an image set are plotted together on the image set.

**Figure 3:**
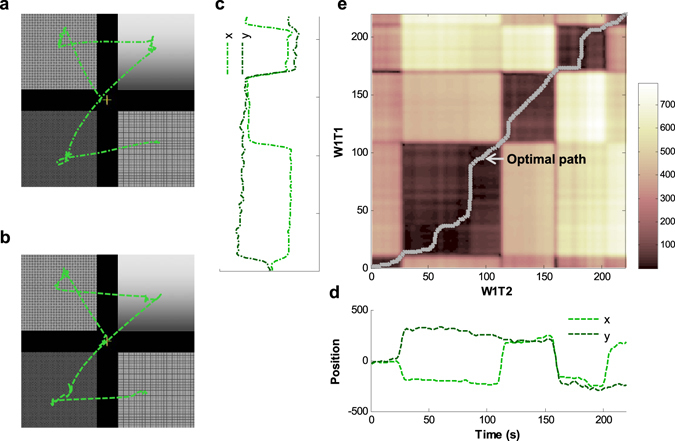
An example of dissimilarity calculation between two scanpaths with DTW. (**a**) A scanpath of subject #20 on image set #47 in session W1T1 and (**b**) one in W1T2. The four images in image set #47 are not shown due to their copyrights; thus, the areas for these images are covered with randomly assigned patterns. The scanpaths in **(a)** and **(b)** can be presented in the time domain as (**c**) and (**d**), respectively. (**e**) A matrix of Euclidean distance between every point of (**c**) and (**d**). Each component is a distance between one point from (**c**) and one from (**d**). The grey path is determined by dynamic programming, and the accumulated distance along this line is defined as dissimilarity between (**c**) and (**d**). In this example, the dissimilarity between (**c**) and (**d**) is 12,353. The possible range of the dissimilarity value is from 0 to 248,900.

Gaze positions during image set observation were extracted from each recorded trial and preprocessed into a continuous scanpath with MATLAB. A scanpath consists of gaze positions at each time point of a trial, and each position was represented on an < x, y > coordinate system with the origin at the centre of the screen. Each image set was presented for two seconds (~240 time points), and the gaze positions of the first 220 time points were extracted. During this period, some of the data positions could be lost due to a subject’s eye blinking or device error. Linear interpolation was used to fill in the missing sections. A smoothing method was used as a low-pass filter in which incremental chunks of six consecutive points were weight-averaged to reduce high frequency noise. Unfortunately, signals in some trials suffered too much data loss and the interpolation was meaningless for those signals. Signals with more than 20% of their data points missing in either x or y coordinates were regarded as invalid eye movement data and discarded. On average, signals in 13.6 (*s*.*d*. = 22.2) of 110 trials were invalid in a session (*n* = 324 sessions). These signals were neither preprocessed nor analysed. After preprocessing, each signal from a trial was considered to be a scanpath (or an invalid scanpath from an invalid signal). Scanpaths of each session were assigned indices corresponding to image sets (1 to 110). Scanpaths induced by the same image set were compared, i.e., only scanpaths with the same index were compared. Examples of preprocessed scanpaths are represented in a 2D coordinate system (Figs 2 and 3a and b) and in the time domain (Fig. 3c and d).

### Pairwise scanpath comparisons and recording time intervals

To observe scanpath idiosyncrasies, each image set’s scanpaths were subjected to pairwise comparisons. Scanpaths from Free and Task sessions were compared separately, and each pair of scanpaths was assigned to a group (among three) according to the following recording time intervals between the two scanpaths: “thirty minutes” (30 M, e.g., time interval between scanpaths of W1T1 and those of W1T2 sessions), “one week” (1 W, e.g., between W1T1 and W2T1), and “two weeks” (2 W, e.g., between W1T1 and W3T1). Dissimilarities between scanpath pairs were analysed according to these time intervals.

Dissimilarity in each scanpath pair was achieved with dynamic time warping (DTW). While a sequence of spatial attention shifts may be consistent, the tempo of these shifts may be relatively less consistent for any subject over repeated exposure to the same stimuli^34, 35^. To align scanpaths according to attention shifting sequences using more than the speed of the shifts, we used DTW. DTW compares two signals by aligning them in the time domain with dynamic programming. This technique was first used for matching speech waveforms for speech recognition by warping samples into an optimal match by minimizing temporal dissimilarities in order to cope with differences in speaking rates^36, 37^. In the present study, DTW warped and aligned pairs of scanpaths and simultaneously retained more fixation duration information than other scanning sequence matching methods, such as string-edit^38, 39^ and ScanMatch^40^. Dissimilarity values were calculated by summing the Euclidean distances between aligned data points (an example is shown in Fig. 3). We modified publicly provided codes^41^, which originated with the project in a reference^37^.

### Statistics

Every *t*-test that we report is one-sided (or right-sided) test. Those *t*-tests without the description of paired/unpaired are all unpaired *t*-tests, and we used a method for unequal sample size and unequal variances for unpaired *t*-tests. Many average values in this study were achieved with large samples; thus, we present Cohen’s *d*s as effect sizes for average comparisons without *t*-tests or with *t*-tests for support. Standardized differences between two means were demonstrated with Cohen’s *d*s. For Cohen’s *ds*, we calculated pooled standard deviations with a method for different sample sizes and variances between two groups^42^.

### Data availability

The data from our experiment are available on request from the corresponding author. The data are not publicly available according to the policy protecting subjects’ personal data.

## Results

In this section, the idiosyncrasies of scanpaths on the same image sets and their temporal robustness were observed under various conditions. The dissimilarities in scanpath pairs were analysed according to the time intervals described in the Materials and Methods section. First, the dissimilarity values from within- and between-subject comparisons were determined. Next, scanpaths were divided into groups with respect to image set contents, and the effects of the contents on scanpath idiosyncrasies were investigated. Each subject’s scanpath distinctiveness was also confirmed with subject identification. These steps of analysis were then conducted on an extended dataset, including scanpaths recorded during an additional experiment, which was performed one year after the original experiment.

### Scanpath dissimilarities

First, to determine whether scanpaths from the same person are more similar to each other than to those from different people, we compared scanpaths within and between subjects. However, subjects’ preferences were compared (Supplementary Fig. S1) before the scanpath comparisons. From the results, we can infer that the subject pool in our study has distinctive preferences regarding the visual stimuli used in our experiment and shares a small portion of common preferences.

**Figure 4:**
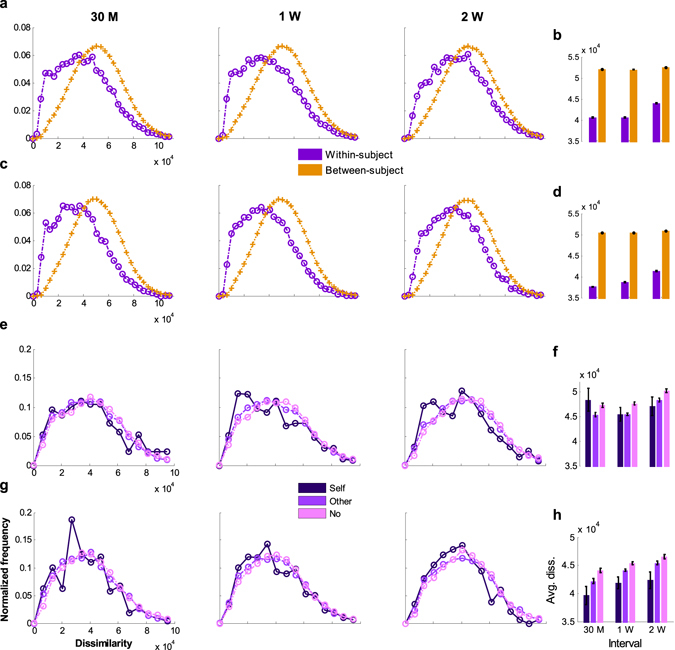
Distributions of scanpath dissimilarity values. The frequency distributions of within- and between-subject scanpath pairs plotted according to the dissimilarity values for (**a**) Free (the number of valid within-subject pairs: *n*_30M_ = 7,122, *n*_1W_ = 18,857, and *n*_2W_ = 9,650 and between-subject pairs: *n*_30M_ = 357,727, *n*_1W_ = 471,825, and *n*_2W_ = 243,674) and (**c**) Task sessions (the number of valid within-subject pairs: *n*_30M_ = 7,009, *n*_1W_ = 18,451, and *n*_2W_ = 9,601 and between-subject pairs: *n*_30M_ = 352,705, *n*_1W_ = 464,890, and *n*_2W_ = 240,604). (**b**) The overall mean dissimilarity of each distribution in (**a**) and (**d**) that of each distribution in (**c**). (**e**) The frequency distributions of within-subject scanpath pairs plotted according to the dissimilarity values of each trial group for Free (the number of pairs from the self-face group: *n*_30M_ = 195, *n*_1W_ = 518, and *n*_2W_ = 260, from the other-face group: *n*_30M_ = 3,725, *n*_1W_ = 9,872, and *n*_2W_ = 5,022, and from the no-face group: *n*_30M_ = 3,202, *n*_1W_ = 8,467, and *n*_2W_ = 4,368) and (**g**) Task sessions (the number of pairs from the self-face group: *n*_30M_ = 190, *n*_1W_ = 505, and *n*_2W_ = 264, from the other-face group: *n*_30M_ = 3,668, *n*_1W_ = 9,658, and *n*_2W_ = 5,012, and from the no-face group: *n*_30M_ = 3,151, *n*_1W_ = 8,288, and *n*_2W_ = 4,325). (**f**) The overall mean dissimilarity of each distribution in (**e**) and (**h**) that of each distribution in (**g**). The sum of each distribution is 1. The error bars in (**b**), (**d**), (**f**), and (**h**) represent standard errors.

Each image set’s scanpaths were compared for every combination of pairs, and dissimilarity values from DTW of valid scanpath pairs were divided into within-subject and between-subject comparison pairs (see the Supplementary Text). The distributions of the dissimilarities were plotted according to the recording time intervals (Fig. 4a–d). The average dissimilarity values of within-subject scanpath pairs were smaller than those of between-subject pairs (Cohen’s *d* = 0.58 for 30 M, 0.58 for 1 W, and 0.44 for 2 W interval with Free and Cohen’s *d* = 0.70 for 30 M, 0.64 for 1 W, and 0.51 for 2 W interval with Task condition). Among within-subject scanpath pairs, pairs with longer time intervals were likely to be less similar than those with shorter intervals (between 30 M and 2 W: Cohen’s *d* = 0.17 for Free and 0.20 for Task condition), whereas among between-subject pairs, this tendency was not observed (between 30 M and 2 W: Cohen’s *d* = 0.02 for both Free Task condition). Although within-subject dissimilarities in scanpaths increased over time, scanpaths from different subjects did not become more similar.

Additionally, differences in the dissimilarity distributions were compared with Kullback-Leibler divergence (KL divergence), which is a measure of the deviation of one probability distribution from another distribution^43^. KL divergence is zero when two distributions are exactly the same, a larger KL value indicates that two distributions diverge. According to the KL divergence results (Supplementary Table S2), within-subject scanpath dissimilarity distributions were more different from between-subject dissimilarity distributions for Task condition; thus, idiosyncrasies in scanpaths were more pronounced during Task than during Free sessions.

In this analysis, dissimilarities between scanpaths were measured with DTW, and the dissimilarity values within and between subjects were compared with statistical tests and KL divergences. Statistical test and KL divergence results both supported relatively smaller dissimilarities in within-subject scanpath comparisons, demonstrating the idiosyncrasies of scanpaths measured in our experiment. Moreover, the idiosyncrasies were temporally more consistent with the preference selection task.

### Effects of image set contents on scanpath consistency

To investigate the effects of image set compositions on scanpath consistency, we first defined and analysed the subject distinguishability of image sets. The ratio of average between-subject scanpath dissimilarity values to average within-subject scanpath dissimilarity values was defined as the subject distinguishability of each image set. The image sets used in our experiment were divided based on different criteria. Among these criteria, only one criterion showed a significant effect on distinguishability, which was the existence of human face images (Supplementary Figs S2–4). Image sets including more than one human face evoked more robust idiosyncratic scanpaths than those without human faces. According to our interpretation, these results indicate that faces in image sets helped construct stronger scanpaths from each subject, since humans have developed the extraordinary ability of processing faces^44, 45^. However, we did not find an effect of the number of faces in an image set.

Among the face images, self-face images are considered as being more special to each subject. To investigate the effects of self-face images on scanpaths over time, within-subject scanpath dissimilarity values were divided into the following different trial groups: “self-face” (3 trials in each session), “other-face” (57 trials in each session), and “no-face” (50 trials in each session) groups. The trial indices for self-face images and other-face images were different for each subject. Values for each group for within-subject pairing were observed with their distributions (Fig. 4e–h).

Within-subject dissimilarities from the self-face group were smaller than those from the other groups in most cases (for details, see Supplementary Table S3), and the differences were relatively smaller between those from the other- and no-face groups. These differences between the distributions of the three group were also supported by KL divergence analysis (Supplementary Table S4). Ultimately, within-subject scanpaths were most robust on image sets including self-faces, especially during Task sessions.

### Subject identification with scanpaths

To better assess the relative consistency of an individual’s scanpaths over time, we adopted methods from research on biometrics^46^ (see the Supplementary Text). Sessions with identity information were labelled “development sessions” and sessions to be identified were labelled “evaluation sessions”. We observed subject identification results with thirty development-evaluation compositions in total, which can also be split into the three time intervals (Supplementary Table S5). Before assessing the scanpaths, we compared a sequence of preferred image indices from each session to each other and identified subjects with up to 88.3% accuracy with 30 M development-evaluation time interval. This result confirms the distinctiveness in each subject’s preferences during our experiment.

**Figure 5:**
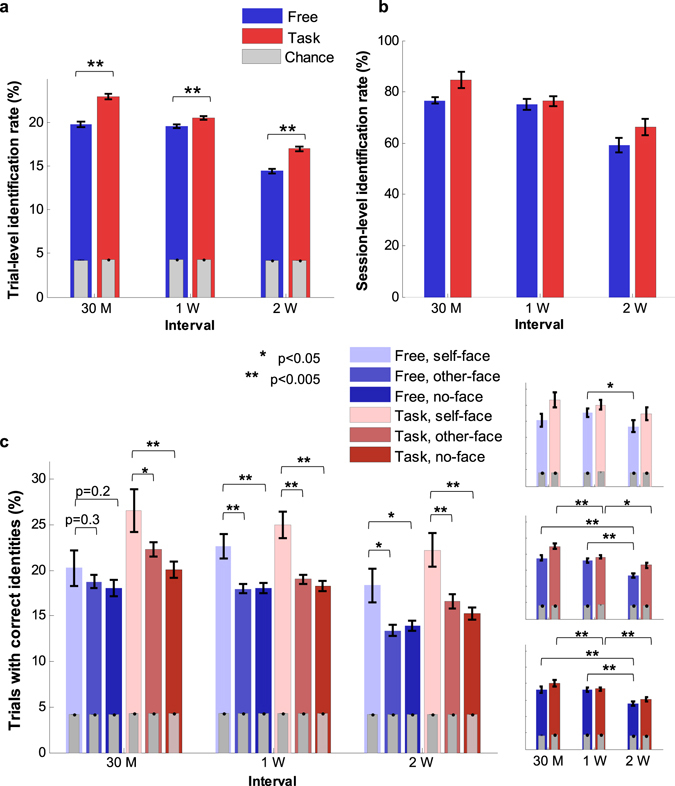
Subject identification performances. (**a**) Average trial-level subject identification rate for each development-evaluation interval (the number of trials for each session condition: *n*_30M_ = 660, *n*_1W_ = 1,760, and *n*_2W_ = 880). Average identification rates with respect to chance levels are compared for different session types and development-evaluation intervals. (**b**) Average session-level subject identification rate for each session type and development-evaluation interval (the number of sessions for each session condition: *n*_30M_ = 6, *n*_1W_ = 16, and *n*_2W_ = 8). (**c**) The ratio of trials that provided correct subject identification decisions in each trial group and for each development-evaluation interval (the number of samples from the self-face group: *n*_30M_ = 148 for Free and 146 for Task, *n*_1W_ = 404 for Free and 390 for Task, and *n*_2W_ = 196 for Free and 200 for Task conditions, from the other-face group: *n*_30M_ = 162 for Free and 160 for Task, *n*_1W_ = 432 for both Free and Task, and *n*_2W_ = 216 for both conditions, and from the no-face group: *n*_30M_ = 162 for both, *n*_1W_ = 430 for both, and *n*_2W_ = 216 for Free and 214 for Task conditions). These results are compared with respect to groups and development-evaluation time intervals. The error bars in (**a**)–(**c**) represent standard errors. The sample numbers for different trial groups and session conditions in (**c**) are not exactly the same due to invalid scanpaths.

During subject identification with scanpaths, each scanpath was identified with the nearest neighbouring^47^ scanpath as determined by DTW, and this process was labelled “trial-level subject identification”. Each scanpath from the evaluation sessions was compared with the corresponding image set’s scanpaths from the development sessions, and 110 trial-level identification rates were determined for each development-evaluation composition (Supplementary Table S6). For every composition, trial-level subject identification rates were significantly higher than chance levels (paired one-sided *t*-test, *t*(109) > 10, *P* < 0.001 for all 30 Free and 30 Task compositions, and normalities were tested with Kolmogorov-Smirnov test (*P* > 0.13 except a composition with *P* = 0.009)). Those identification rates were then grouped according to the development-evaluation time interval (Fig. 5a and Supplementary Table S7). Task sessions demonstrated higher identification rates with respect to chance levels (a chance level was subtracted from each identification rate) than Free sessions for all development-evaluation intervals (paired one-sided *t*-test, *t*(659) = 7.00, *P* < 0.001, Cohen’s *d* = 0.37 for 30 M, *t*(1759) = 3.27, *P* < 0.001, Cohen’s *d* = 0.10 for 1 W, *t*(879) = 6.98, *P* < 0.001, Cohen’s *d* = 0.32 for 2 W). As the intervals between development and evaluation data increased, trial-level identification rates with respect to chance levels decreased; identification rates from 30 M were higher than those from 1 W (Cohen’s *d* = 0.29 for Task condition), and rates from 1 W were higher than those from 2 W (Cohen’s *d* = 0.61 for Free and 0.40 for Task condition). However, we did not observe a significant difference between identification rates from 30 M and those from 1 W for Free condition (Cohen’s *d* = 0.03).

To certify the benefit of DTW, scanpaths were also compared without time warping. Without time warping, trial-level identification rates were significantly lower than those with DTW in all cases (Supplementary Fig. S5 and Table S8). These results indicate that comparing scanpaths with eye movement velocity information suppressed emphasizes within-subject eye movement similarity, confirming the advantage of using DTW for scanpath analysis in this context.

Trial-level subject identification results were compiled during “session-level subject identification”, and an identification decision was reached by majority vote^48^ for each session. In the majority voting method, scanpaths that were identified as each candidate identity were counted for each evaluation session, and the session was identified as an identity with the maximum counts. A session-level identification rate for each development-evaluation composition was determined (Supplementary Table S9), and an average rate for each interval was calculated (Fig. 5b and Supplementary Table S10). Task sessions outperformed Free sessions in most cases as hypothesized. Although statistical confirmation of these results was ineffective due to the small number of samples, the results indicate that the preference selection task induced greater scanpath consistency over time. We could increase subject identification performances with more development data for each evaluation session (Supplementary Text and Fig. S6).

With dissimilarities between scanpaths, we conducted subject identification at both trial and session levels. In our identification method, a scanpath could be correctly identified if it was most similar to a scanpath of the genuine identity. Therefore, identification rates can effectively indicate the similarities between scanpaths from the same subjects compared with those from different subjects. The results of this section also demonstrated that the preference selection task contributed to the robustness of idiosyncrasies in scanpaths.

### Effects of image set contents on subject identification

The effect of self-faces on scanpath robustness was also analysed with the subject identification method. The ratio of correct subject identification decisions to all valid trials was determined for each evaluation session and each image group and presented for each development-evaluation interval (Fig. 5c). Trials from the self-face group correctly identified subjects at higher rates with respect to chance levels than those from other groups in most cases, and the results from the other- and no-face groups were similar (for details, see Supplementary Table S11). Moreover, decreases in correct identification over longer development-evaluation intervals were less significant for trials involving self-face images (Supplementary Table S12). The results from this analysis are consistent with the observation of the previously mentioned within-subject scanpath dissimilarities. Each subject’s scanpaths induced by self-referential stimuli were more consistent in time than those induced by other stimuli.

### Comparisons of scanpaths with a longer time interval

**Figure 6:**
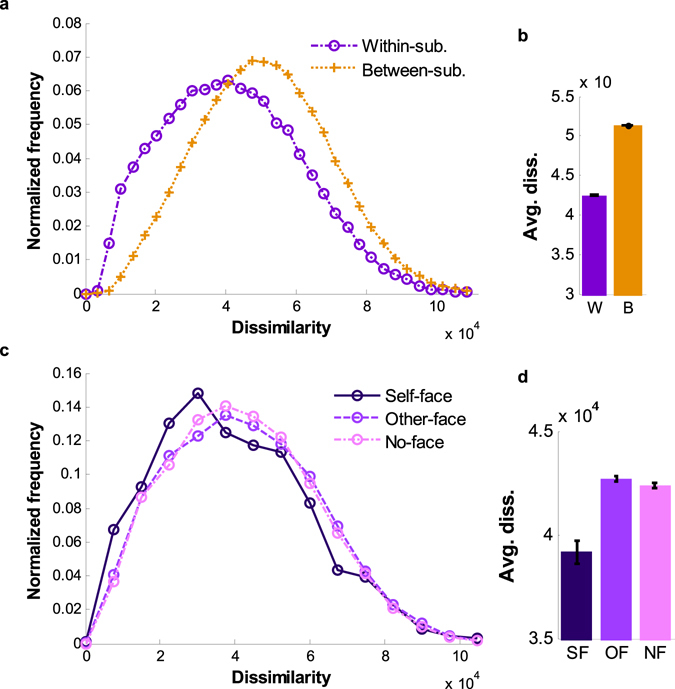
The frequency distributions of dissimilarity values between scanpath pairs with 1 Y interval. (**a**) The frequency distributions of within-subject (*n* = 42,206) and between-subject scanpath pairs (*n* = 551,851) plotted according to dissimilarity values. (**b**) The overall mean dissimilarity of each distribution in (**a**) (W: within-subject scanpath pairs, B: between-subject scanpath pairs). Similar to results from the original data, dissimilarity values from within-subject scanpath pairs are more distributed on the smaller values than those from between-subject pairs (Cohen’s *d* = 0.45). KL divergence from the distribution of between-subject scanpath dissimilarity values (${{\rm{SP}}}_{{\rm{B}}}$) to that of within-subject (${{\rm{SP}}}_{{\rm{W}}}$) is 0.12 (namely, ${{\rm{D}}}_{{\rm{KL}}}({{\rm{SP}}}_{{\rm{B}}}\parallel {{\rm{SP}}}_{{\rm{W}}})=0.12$). (**c**) The frequency distributions of within-subject scanpath pairs plotted according to the dissimilarity values of each trial group (*n*_self-face_ = 1,216, *n*_other-face_ = 22,278, and *n*_no-face_ = 18,712). (**d**) The overall mean dissimilarity of each distribution in (**c**) (SF: self-face, OF: other-face, NF: no-face trial group). Within-subject dissimilarity values are smaller for the self-face trial group than those for the other-face (Cohen’s *d* = 0.16) and no-face trial groups (Cohen’s *d* = 0.15), and the differences in the distributions can be presented as ${{\rm{D}}}_{{\rm{K}}{\rm{L}}}({{\rm{S}}{\rm{P}}}_{{\rm{S}}{\rm{F}}}\parallel {{\rm{S}}{\rm{P}}}_{{\rm{O}}{\rm{F}}})=0.02\,{\rm{a}}{\rm{n}}{\rm{d}}\,{{\rm{D}}}_{{\rm{K}}{\rm{L}}}({{\rm{S}}{\rm{P}}}_{{\rm{S}}{\rm{F}}}\parallel {{\rm{S}}{\rm{P}}}_{{\rm{N}}{\rm{F}}})=0.05$. Although the values for effect size are not strongly supportive, the distributions from the other-face trial group and no-face are relatively similar to each other (Cohen’s *d* = 0.02 and ${{\rm{D}}}_{{\rm{KL}}}({{\rm{SP}}}_{{\rm{OF}}}\parallel {{\rm{SP}}}_{{\rm{NF}}})=0.003$). The scanpaths of the 14 subjects who participated in the additional experiment were observed. The error bars in (**b**) and (**d**) represent standard errors.

Thus far, our observation indicates that the idiosyncrasies of individual scanpaths can be maintained for two weeks. However, we wondered if the idiosyncrasies could still be observed after a few months. Therefore, we recorded additional Task sessions approximately one year after the original experiment with a subset (14 subjects) of the original subjects (see the Supplementary Text for details). The time interval between the scanpaths of the whole original data set and those of the additional data was defined as “one year” (1 Y), and we extended our analyses on this extended data set. The same data processing and analysis methods were performed on these additional Task sessions. Dissimilarity value distributions (Fig. 6) and identification rates (Supplementary Fig. S7) were observed at an approximate 1 Y interval, and the results demonstrated that individual scanpath consistency remains even after one year.

## Discussion

This study complements previous research on the consistencies of idiosyncratic scanpaths by investigating factors that affect their temporal robustness. We designed an experiment to record subjects’ scanpaths induced by attention shifts over images containing different contents, repeatedly conducted the experiment, and longitudinally observed the scanpaths. Every pair of scanpaths was compared, and their dissimilarity values were analysed with DTW. Additionally, we attempted subject identification with the scanpaths. Our results indicated that scanpaths from the same individual are more similar to each other than to those from different individuals, and they are in agreement with other studies that demonstrated within-subject scanpath similarities^12, 13, 20^ and identified subjects by their eye movements^15, 16^. However, we extended the findings by analysing the temporal robustness of the idiosyncratic scanpaths representing individuals’ attention shifts. The similarities between a subject’s scanpaths decreased with longer recording time intervals. However, the distributions of within-subject scanpath dissimilarities remained distinctive from those of between-subject scanpaths dissimilarities, and a single scanpath from a specific subject was identified as the correct subject by comparison with another scanpath measured up to a year previously. These results demonstrated the idiosyncrasies of individual eye movements and their temporal consistencies with regard to the same visual stimuli. The idiosyncrasies were more pronounced and robust with scanpaths during the preference selection task. Additionally, we demonstrated that subjects’ self-facial images were beneficial for maintaining the idiosyncrasies. According to our interpretation of the results, the presence of self-referential images and the preference selection task attract uniquely identifying attention from each individual with stronger top-down modulation, thus stabilize scanpaths over time.

Eye tracking is becoming more prevalent with rapidly growing ubiquitous technologies. Studies have introduced feasible applications using human eye movement tracking, such as user authentication^15, 16^ or monitoring systems^18^. Our research provides scientific support for those applications by analysing the idiosyncrasies of individuals’ scanpaths over time and revealing the factors that contribute to the consistent yet idiosyncratic scanpaths representing attention shifts. Future research is required to address the extent to which other forms of self-referential stimuli or conditions potentially influence individual eye movement consistency.

## Electronic supplementary material

Supplementary Information

## References

[1] Weichselgartner E, Sperling G (1987). Dynamics of automatic and controlled visual attention. Science.

[2] Kowler E, Anderson E, Dosher B, Blaser E (1995). The role of attention in the programming of saccades. Vision Res..

[3] Peterson M, Kramer A, Irwin D (2004). Covert shifts of attention precede involuntary eye movements. Percept. Psychophys..

[4] Theeuwes J, Belopolsky AV (2012). Reward grabs the eye: oculomotor capture by rewarding stimuli. Vision Res..

[5] Yarbus, A. L. Eye movements during perception of complex objects in *Eye Movements and Vision* 171–211 (Plenum Press, 1967).

[6] Borji A, Itti L (2014). Defending Yarbus: eye movements reveal observers’ task. J. Vision.

[7] Sugano Y, Ozaki Y, Kasai H, Ogaki K, Sato Y (2014). Image preference estimation with a data-driven approach: a comparative study between gaze and image features. Eye Movement Res..

[8] Zangemeister WH, Sherman K, Stark L (1995). Evidence for a global scanpath strategy in viewing abstract compared with realistic images. Neuropsychologia.

[9] Field M, Cox WM (2008). Attentional bias in addictive behaviors: a review of its development, causes, and consequences. Drug Alcohol Depend..

[10] Castellanos EH (2009). Obese adults have visual attention bias for food cue images: evidence for altered reward system function. Int. J. Obes..

[11] Mogg K, Bradley BP, Field M, De Houwer J (2003). Eye movements to smoking‐related pictures in smokers: relationship between attentional biases and implicit and explicit measures of stimulus valence. Addiction.

[12] Noton D, Stark L (1971). Scanpaths in eye movements during pattern perception. Science.

[13] Josephson S, Holmes ME (2002). Visual attention to repeated internet images: testing the scanpath theory on the world wide web. Proc. ETRA.

[14] Itti L, Koch C (2001). Computational modelling of visual attention. Nat. Rev. Neurosci..

[15] Cantoni V, Faldi C, Nappi M, Porta M, Ricco D (2015). GANT: gaze analysis technique for human identification. Pattern Recogn..

[16] Rigas I, Komogortsev O (2014). Biometric Recognition via Probabilistic Spatial Projection of Eye Movement Trajectories in Dynamic Visual Environments. IEEE Trans. Inf. Forensics Security.

[17] Pieters R, Warlop L (1999). Visual attention during brand choice: the impact of time pressure and task motivation. Int. J. Res. Mark..

[18] Ji Q, Zhu Z, Lan P (2004). Real-time nonintrusive monitoring and prediction of driver fatigue. IEEE Trans. Veh. Technol..

[19] Judd T, Durand F, Torralba A (2011). Fixations on low-resolution images. J. Vision.

[20] Walker-Smith G, Gale A, Findlay J (1977). Eye movement strategies involved in face perception. Perception.

[21] Park J, Shimojo E, Shimojo S (2010). Roles of familiarity and novelty in visual preference judgments are segregated across object categories. Proc. Natl. Acad. Sci. USA.

[22] Shimojo S, Simion C, Shimojo E, Scheier C (2003). Gaze bias both reflects and influences preference. Nat. Neurosci..

[23] Mitsuda T, Glaholt MG (2014). Gaze bias during visual preference judgements: effects of stimulus category and decision instructions. Vis. Cogn..

[24] Glaholt MG, Reingold EM (2011). Eye movement monitoring as a process tracing methodology in decision making research. J. Neurosci. Psychol. Econ..

[25] Wolford G, Morrison F (1980). Processing of unattended visual information. Mem. Cognition.

[26] Devue C, Brédart S (2008). Attention to self-referential stimuli: can I ignore my own face?. Acta Psychol..

[27] Althoff RR, Cohen NJ (1999). Eye-movement-based memory effect: a reprocessing effect in face perception. J. Exp. Psychol.-Learn. Mem. Cogn..

[28] Barton JJ, Radcliffe N, Cherkasova MV, Edelman J, Intriligator JM (2006). Information processing during face recognition: the effects of familiarity, inversion, and morphing on scanning fixations. Perception.

[29] Phillips PJ, Moon H, Rizvi SA, Rauss PJ (2000). The FERET evaluation methodology for face-recognition algorithms. IEEE Trans. Pattern Anal. Mach. Intell..

[30] Russakovsky O (2015). Imagenet large scale visual recognition challenge. Int. J. Computer Vision.

[31] Winkler S, Ramanathan S (2013). Overview of eye tracking datasets. Proc. QoMEX.

[32] Duchowski, A. Visual psycolphysics in *Eye Tracking Methodology*: *Theory and Practice (2*^*nd*^*ed*.*)* 29–39 (Springer Science & Business Media, 2007).

[33] Tobii Technology AB, User manual–Tobii Studio, Manual Ver. 3.2, Rev A. 11 (2012).

[34] Anderson NC, Anderson F, Kingston A, Bischof WF (2015). A comparison of scanpath comparison methods. Behav. Res. Meth..

[35] Pieters R, Rosbergen E, Wedel M (1999). Visual attention to repeated print advertising: a test of scanpath theory. J. Marketing Res..

[36] Sakoe H, Chiba S (1978). Dynamic programming algorithm optimization for spoken word recognition. IEEE Trans. Acoust., Speech, Signal Process..

[37] Turetsky, R. J. & Ellis, D. P. Ground-truth transcriptions of real music from force-aligned midi syntheses. *Proc*. *ISMIR 2003*, 135–141 (2003).

[38] Levenshtein VI (1966). Binary codes capable of correcting deletions, insertions, and reversals. Soviet Physics Doklady.

[39] Brandt SA, Lawrence WS (1997). Spontaneous eye movements during visual imagery reflect the content of the visual scene. J. Cognitive Neurosci..

[40] Cristino F, Mathôt S, Theeuwes J, Gilchrist ID (2010). ScanMatch: a novel method for comparing fixation sequences. Behav. Res. Meth..

[41] Ellis, D. Dynamic Time Warp (DTW) in Matlab. *Columbia University* http://www.ee.columbia.edu/~dpwe/resources/matlab/dtw/ (2003).

[42] Cohen, J. *Statistical power analysis for the behavioural sciences (Rev. ed.)* (Academic Press Inc., 1977).

[43] Kullback, S. *Information Theory and Statistics*, (Dover Publications Inc., 1968).

[44] Little AC, Jones BC, DeBruine LM (2011). Facial attractiveness: evolutionary based research. Phil. Trans. R. Soc. B.

[45] Fuhrmann D (2016). Perception and recognition of faces in adolescence. Sci. Rep..

[46] Jain AK, Ross A, Prabhakar S (2004). An introduction to biometric recognition. IEEE Trans. Circuits, Syst. Video Technol..

[47] Cover TM, Hart PE (1967). Nearest neighbor pattern classification. IEEE Trans. Inf. Theory.

[48] Polikar R (2006). Ensemble based systems in decision making. IEEE Circuits Syst. Mag..

