# Supplementary material for: Temporally Robust Eye Movements through Task Priming and Self-referential Stimuli
Source: Sci Rep. 2017 Aug 3;7:7257. doi: 10.1038/s41598-017-07641-7 (PMC5543141; doi:10.1038/s41598-017-07641-7)
Supplement: Supplementary file 1 — Supplementary Information [file 41598_2017_7641_MOESM1_ESM.pdf]

Supplementary Information for

Temporally Robust Eye Movements through Task Priming and Self-referential Stimuli

Eun-Soo Jung, Dong-Gun Lee, Kyeongho Lee, Soo-Young Lee

Correspondence to: sy-lee@kaist.ac.kr

This file includes:

Figs. S1 to S7
Tables S1 to S12
Supplementary text
References (*1-48*)

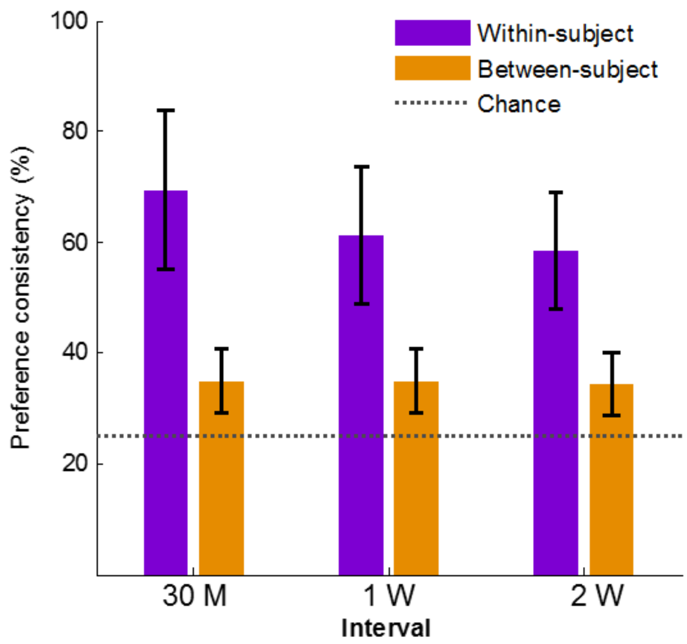

Fig. S1. Comparisons of subjects' preferences.

An average preference coherence of session pairs for each time interval. During Task sessions, each subject selected a preferred image in each trial. A sequence of selected images was generated for each Task session, and every pair of these sequences was compared. For each pair of sequences, a ratio of the same preferences out of 110 matches represented the preference coherence. Average within-subject preference coherence (n_{30M}=81, n_{1W}=216, and n_{2W}=108) was observed with each time interval and was compared with average between-subject preference coherence (n_{30M}=4,212, n_{1W}=5,626, and n_{2W}=2,808) as a control. Additionally, coherences were compared with respect to different time intervals. The ratio of the same preferences is significantly higher for within-subject pairs than for between-subject pairs. Session pairs with larger time intervals show smaller within-subject preference coherence (Cohen's d=0.64 between 30 M and 1 W, and 0.23 between 1 W and 2 W intervals), which indicates that subjects' preferences vary with time. The preferred images of each subject can change over time; thus, pairs of preference sequences with larger time intervals share relatively less coherence for each subject. Still, these pairs have more coherence than those from between-subject comparisons. Between-subject session pairs do not show a strong relationship between preference coherence and time interval (Cohen's d=0.02 between 30 M and 1 W, 0.10 between 1 W and 2 W intervals), and their average coherences are above chance level. Error bars represent standard deviations.

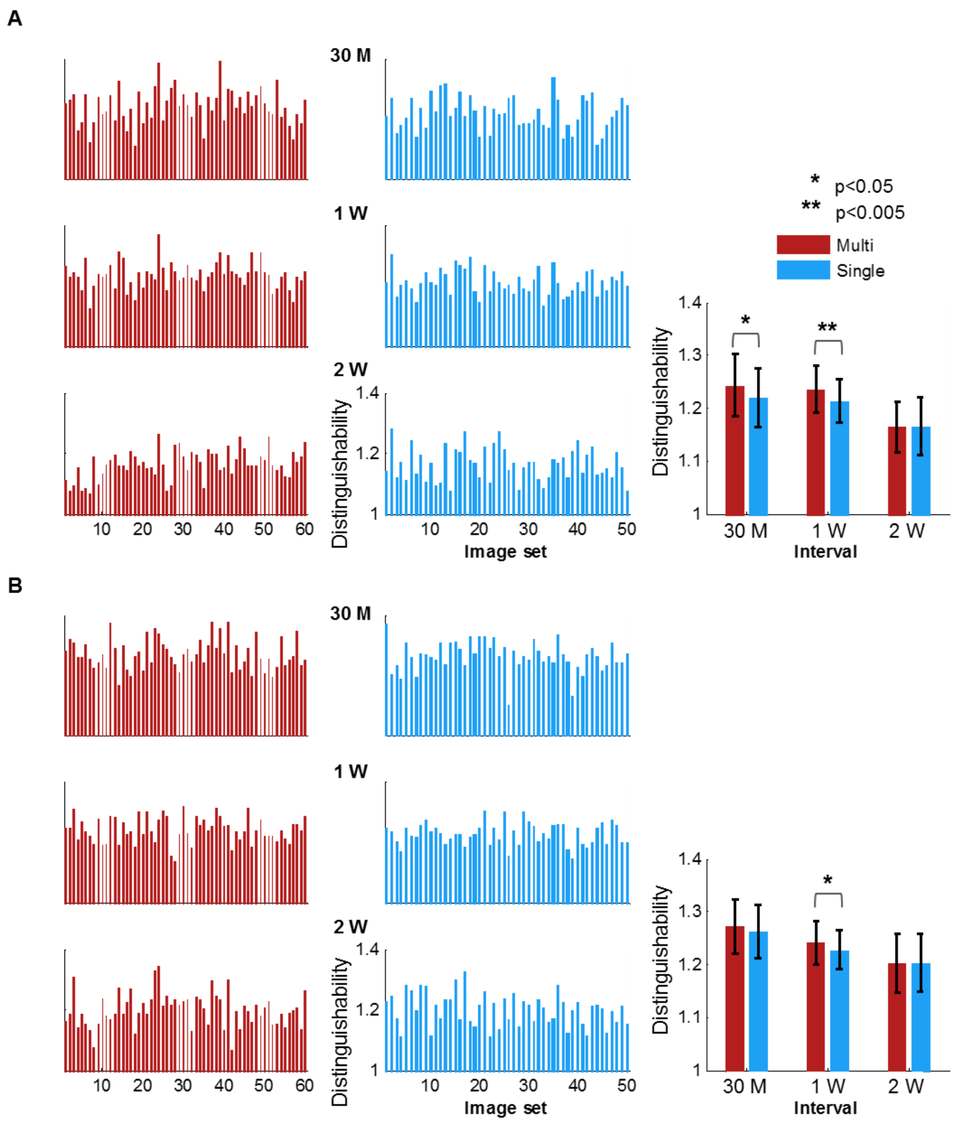

Fig. S2. Observations of subject distinguishability regarding the number of image categories in an image set.

Subject distinguishability of image sets divided into "multi-category" and "single-category" groups. Each image set in the single-category group (total 60 image sets) consists of four images in a single category, whereas each image set in a multi-category group (total 50 image sets) contains four images that cannot be classified into one category. Subject distinguishability for the two groups is compared for **(A)** Free and **(B)** Task conditions. The average distinguishability of the multi-category group is significantly higher for 30 M interval (one-sided *t*-test, $t(106.9)=2.13$, $P=0.018$, Cohen's $d=0.40$) and 1 W interval ($t(106.6)=2.75$, $P=0.003$, Cohen's $d=0.52$) under Free condition and for 1 W interval ($t(106.9)=2.13$, $P=0.018$, Cohen's $d=0.40$) under Task condition. However, the remaining cases do not exhibit this tendency (2 W: $t(98.9)=-0.07$, $P=0.528$, Cohen's $d=0.01$ for Free, and 30 M: $t(104.5)=0.86$, $P=0.195$, Cohen's $d=0.16$ and 2 W: $t(105.8)=-0.09$, $P=0.535$, Cohen's $d=0.02$ for Task). Overall, our conclusion is that these results do not support the dependency of subject distinguishability on the compositions of image sets. Error bars represent standard deviations, and normality tests were performed with Kolmogorov-Smirnov test ($P>0.25$ for all groups).

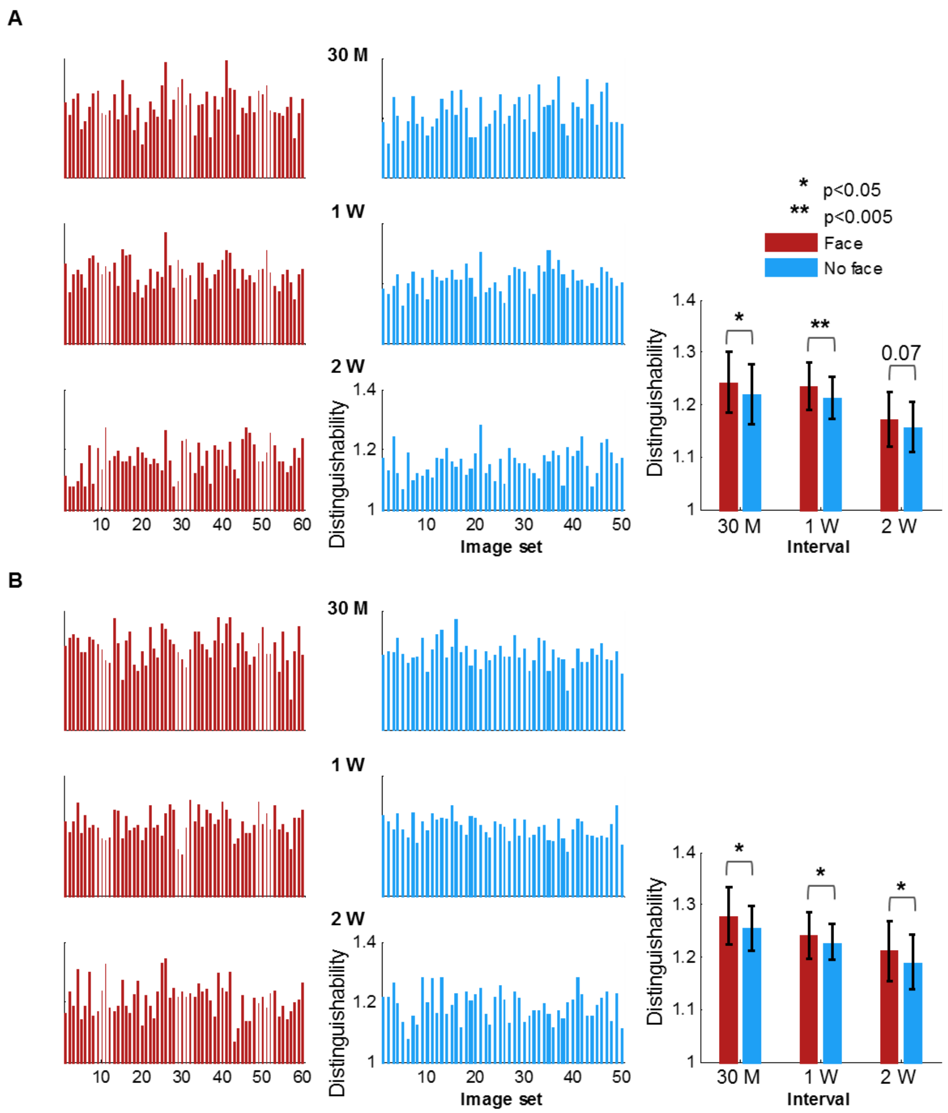

Fig. S3. Observations of subject distinguishability regarding facial images in an image set. The subject distinguishability of image sets divided into "face" and "no-face" groups. There are 60 image sets where each includes at least one human facial image (face group) and 50 image sets with non-facial images (no-face group). The subject distinguishability for the two groups is compared for **(A)** Free and **(B)** Task conditions. The average distinguishability of the image sets that include facial image(s) is significantly higher in most cases (one-sided *t*-test, 30 M: *t*(104.8)=2.05, *P*=0.021, Cohen's *d*=0.39, 1 W: *t*(107.7)=2.76, *P*=0.003, Cohen's *d*=0.52, and 2 W: *t*(107.6)=1.49, *P*=0.070, Cohen's *d*=0.28 for Free condition and 30 M: *t*(107.3)=2.62, *P*=0.005, Cohen's *d*=0.49, 1 W: *t*(107.8)=1.76, *P*=0.040, Cohen's *d*=0.33, and 2 W: *t*(107.4)=2.06, *P*=0.021, Cohen's *d*=0.39 for Task condition). These results indicate that human faces enhance the idiosyncrasies of individual scanpaths. Error bars represent standard deviations, and normality tests were performed with Kolmogorov-Smirnov test (*P*>0.63 for all groups).

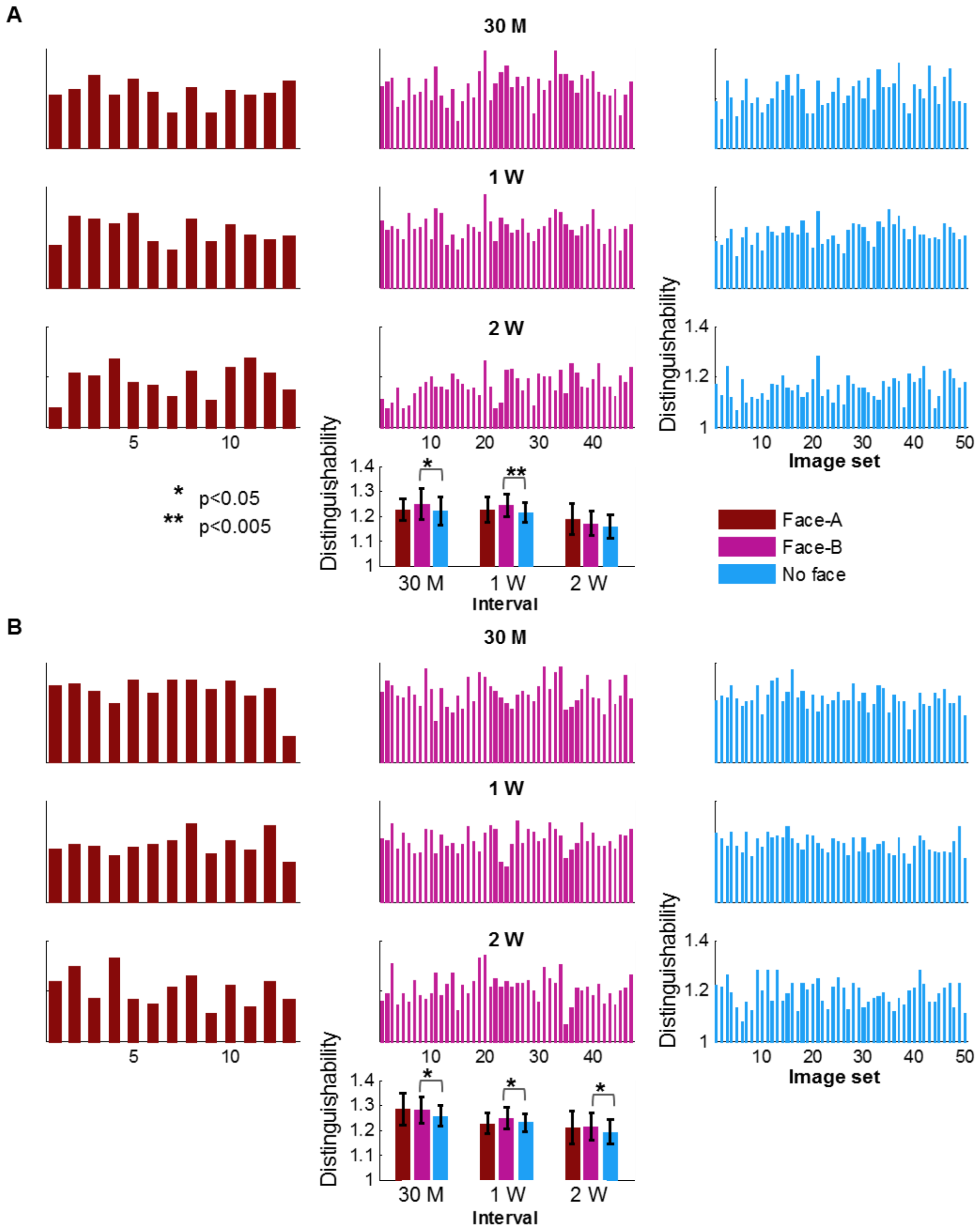

Fig. S4. Observations of subject distinguishability regarding the number of facial images in an image set.

The subject distinguishability of image sets divided into "face-A," "face-B," and "no-face" trial groups. Among 60 image sets including human facial image(s), 13 image sets include only facial images (face-A), and each of the remaining 47 image sets contain at least one facial image and contain at least one non-facial image (face-B). Additionally, 50 image sets contain no facial image at all (no-face). The subject distinguishability for the three groups is compared for **(A)** Free and **(B)** Task conditions. The differences between face-A and face-B are inconsistent. However, the subject distinguishability of face-B was significantly higher than that of the no-face group except for the 2 W interval under Free condition (one-sided *t*-test, 30 M: $t(93.7)=2.30$, $P=0.012$, Cohen's $d=0.47$, 1 W: $t(92.3)=2.99$, $P=0.002$, Cohen's $d=0.61$, and 2 W: $t(93.5)=0.98$, $P=0.164$,

Cohen's $d=0.20$ for Free condition and 30 M: $t(87.52)=2.34$, $P=0.011$, Cohen's $d=0.47$, 1 W: $t(88.0)=2.16$, $P=0.017$, Cohen's $d=0.44$, and 2 W: $t(93.2)=2.06$, $P=0.021$, Cohen's $d=0.42$ for Task condition). Additionally, we can conclude that the number of faces in an image set is not as important as the existence of a face, though the number of samples in face-A group is small for a reliable statistical test. Error bars represent standard deviations, and normality tests were performed with Kolmogorov-Smirnov test ($P>0.40$ for all groups).

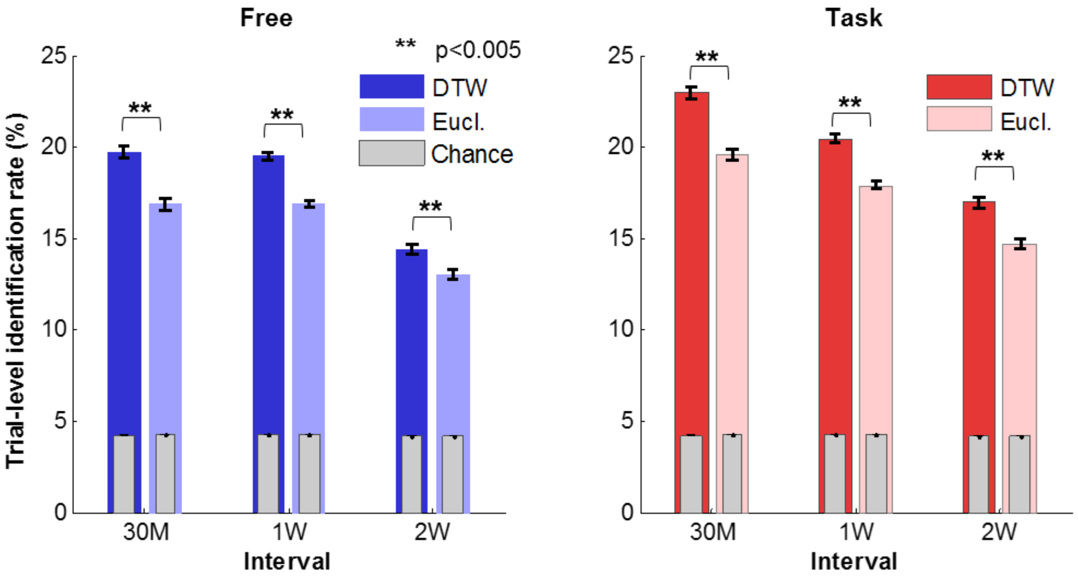

Fig. S5. Trial-level subject identification rates with and without time warping.

When two scanpaths were compared with DTW, gaze points were aligned with time warping. The results without time warping (labelled Euclidean method) are shown and compared with those of DTW. In the Euclidean method, pairs of scanpaths were aligned without time warping, and Euclidean distances between corresponding gaze positions at each time point were summed as a measure of the total dissimilarity. DTW achieves a higher average identification rate (paired one-sided t-test, 30 M: $t(659)=9.00$, $P<0.001$, Cohen's $d=0.34$, 1 W: $t(1759)=13.60$, $P<0.001$, Cohen's $d=0.31$, and 2 W: $t(879)=5.93$, $P<0.001$, Cohen's $d=0.20$ for the Free condition and 30 M: $t(659)=10.51$, $P<0.001$, Cohen's $d=0.40$, 1 W: $t(1759)=12.77$, $P<0.001$, Cohen's $d=0.29$, and 2 W: $t(879)=8.78$, $P<0.001$, Cohen's $d=0.29$ for the Task condition) in every session type and development-evaluation interval. These results indicate that comparing scanpaths with less consideration of eye movement velocity information can more effectively detect within-subject eye movement similarity, confirming the advantage of using time warping for scanpath analysis. Error bars represent standard errors.

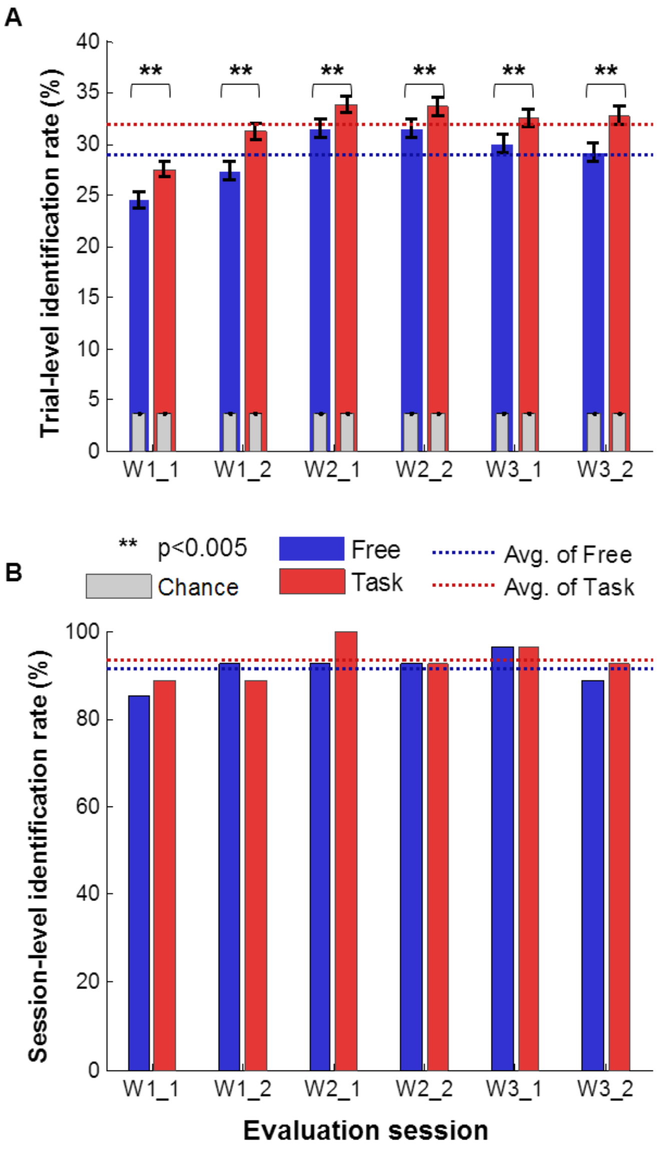

Fig. S6. Identification results with extended development sessions.

(A) Trial-level subject identification rate for each evaluation session (where all the other five sessions are used as development data). Overall, trial-level subject identification performance is improved with respect to the initial results (in Fig. 5a). Again, trial-level subject identification rates within Task sessions are significantly higher than those within Free sessions (paired one-sided *t*-test, $t(109)=2.89$, $P=0.002$, Cohen's $d=0.36$ between W1T1 and W1F1, $t(109)=3.29$, $P<0.001$, Cohen's $d=0.43$ between W1T2 and W1F2, $t(109)=2.12$, $P=0.018$, Cohen's $d=0.26$ between W2T1 and W2F1, $t(109)=1.69$, $P=0.047$, Cohen's $d=0.24$ between W2T2 and W2F2, $t(109)=2.27$, $P=0.013$, Cohen's $d=0.28$ between W3T1 and W3F1, and $t(109)=2.90$, $P=0.002$, Cohen's $d=0.38$ between W3T2 and W3F2). Error bars represent standard errors, and normality tests were performed with Kolmogorov-Smirnov test ($P>0.63$ for all groups). **(B)** Session-level subject identification rate for each evaluation session. Average session-level subject identification rates are improved to 91.4% for Free and 93.2% for Task sessions.

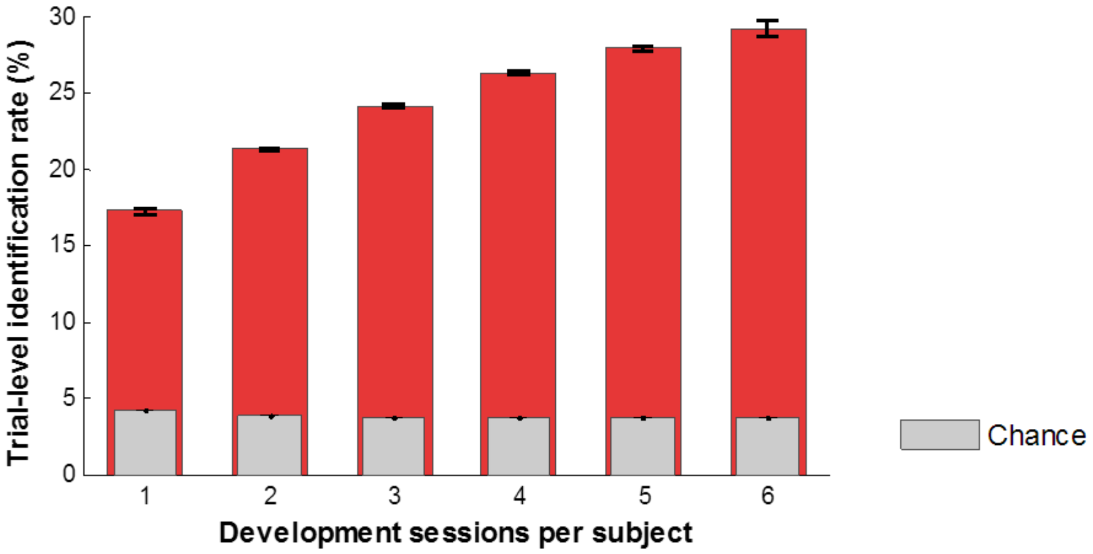

Fig. S7. Identification results for 1 Y time interval between development and evaluation data with various development session combinations.

Task sessions in the original experiment were used as development data with 27 candidate identities, and data from the additional experiment were used for evaluation. When a single session per subject is used as development data, the average trial-level subject identification rate for 1 Y time interval is smaller than that for shorter time intervals; however, more scanpaths are correctly identified as the number of development sessions increase. Overall, we can conclude that idiosyncrasies in individual scanpaths can last even after a year. Error bars represent standard errors.

Table S1. Categories of images used in the experiment.
Images used in the experiment were divided into the categories indicated.

Category	Number of images
Face	120
Cartoon character	30
Place	60
Animal	30
Sport	40
Musical instrument	20
Food	35
Plant	20
Logo	20
Car	20
Cosmetic	15
Brand product	30
Total	440

Table S2. KL divergences between the distributions of within- and between-subject scanpath dissimilarities.

KL divergence between the distribution of within-subject scanpath dissimilarities (SP_W) and the distribution of between-subject scanpath dissimilarities (SP_B) are presented for each time interval.

Free

	**30 M**	**1 W**	**2 W**
$D_{KL}(SP_B \parallel SP_W)$	0.25	0.26	0.14

Task

	**30 M**	**1 W**	**2 W**
$D_{KL}(SP_B \parallel SP_W)$	0.33	0.27	0.18

Table S3. Comparisons of dissimilarity values between trial groups.

The average dissimilarity values of within-subject scanpath pairs, which are divided according to the image set contents, are compared. Cohen's *d* with unequal sample sizes and unequal variances are presented.

Free

	**30 M**	**1 W**	**2 W**
Between other-face and self-face	<0.01	0.11	0.18
Between no-face and self-face	0.11	0.19	0.25
Between no-face and other-face	0.12	0.08	0.07

Task

	**30 M**	**1 W**	**2 W**
Between other-face and self-face	0.10	0.13	0.23
Between no-face and self-face	0.20	0.20	0.29
Between no-face and other-face	0.11	0.06	0.05

Table S4. KL divergences between dissimilarity distributions regarding image set contents. Within-subject scanpath pairs were divided into the self-face ($\mathrm{SP_{SF}}$), other-face ($\mathrm{SP_{OF}}$), and no-face ($\mathrm{SP_{NF}}$) groups. The dissimilarity distributions of the three groups were compared pairwise.

Free

	**30 M**	**1 W**	**2 W**
$\mathrm{D_{KL}(SP_{SF} \parallel SP_{OF})}$	0.031	0.029	0.037
$\mathrm{D_{KL}(SP_{SF} \parallel SP_{NF})}$	0.026	0.041	0.064
$\mathrm{D_{KL}(SP_{OF} \parallel SP_{NF})}$	0.044	0.015	0.014

Task

	**30 M**	**1 W**	**2 W**
$\mathrm{D_{KL}(SP_{SF} \parallel SP_{OF})}$	0.032	0.022	0.055
$\mathrm{D_{KL}(SP_{SF} \parallel SP_{NF})}$	0.054	0.031	0.069
$\mathrm{D_{KL}(SP_{OF} \parallel SP_{NF})}$	0.021	0.004	0.005

Table S5. Development-evaluation time intervals for subject identification.

Every development-evaluation composition and its time interval between development and evaluation data are presented. There are 30 compositions for each session type, including six compositions with a thirty-minute (30 M) interval, sixteen with a one-week (1 W) interval, and eight with a two-week (2 W) interval.

Free sessions

Evaluation data (from each subject) / Development data (from each subject)	W1F1	W1F2	W2F1	W2F2	W3F1	W3F2
**W1F1**		30 M	1 W	1 W	2 W	2 W
**W1F2**	30 M		1 W	1 W	2 W	2 W
**W2F1**	1 W	1 W		30 M	1 W	1 W
**W2F2**	1 W	1 W	30 M		1 W	1 W
**W3F1**	2 W	2 W	1 W	1 W		30 M
**W3F2**	2 W	2 W	1 W	1 W	30 M	

Task sessions

Evaluation data (from each subject) / Development data (from each subject)	W1T1	W1T2	W2T1	W2T2	W3T1	W3T2
**W1T1**		30 M	1 W	1 W	2 W	2 W
**W1T2**	30 M		1 W	1 W	2 W	2 W
**W2T1**	1 W	1 W		30 M	1 W	1 W
**W2T2**	1 W	1 W	30 M		1 W	1 W
**W3T1**	2 W	2 W	1 W	1 W		30 M
**W3T2**	2 W	2 W	1 W	1 W	30 M	

Table S6. Trial-level subject identification rate for every development-evaluation composition.

An average of 110 trial-level subject identification rates (%) are presented for each development-evaluation composition. Standard deviations appear in parentheses.

Free sessions

Dev. \ Eval.	W1F1	W1F2	W2F1	W2F2	W3F1	W3F2
**W1F1**		17.8 (7.7)	19.7 (7.8)	14.2 (6.8)	14.8 (6.6)	12.6 (6.7)
**W1F2**	18.4 (7.5)		20.4 (9.0)	19.2 (8.1)	16.4 (6.9)	16.5 (8.4)
**W2F1**	19.1 (8.2)	20.1 (9.6)		20.2 (9.3)	21.3 (9.1)	17.3 (7.7)
**W2F2**	16.2 (7.6)	19.4 (8.4)	20.4 (9.9)		21.5 (8.8)	22.7 (9.0)
**W3F1**	13.6 (7.4)	15.8 (6.6)	21.9 (9.0)	21.7 (9.0)		21.4 (8.5)
**W3F2**	12.1 (6.9)	13.7 (7.6)	16.0 (8.2)	21.8 (8.6)	20.4 (8.3)	

Task sessions

Dev. \ Eval.	W1T1	W1T2	W2T1	W2T2	W3T1	W3T2
**W1T1**		22.4 (8.0)	19.0 (7.3)	14.6 (7.9)	16.4 (7.8)	16.2 (8.7)
**W1T2**	22.5 (7.9)		23.6 (9.4)	21.0 (9.0)	19.0 (8.4)	18.3 8.4)
**W2T1**	19.3 (7.0)	23.2 (9.9)		23.0 (8.3)	21.5 (8.7)	19.0 (7.8)
**W2T2**	14.1 (7.3)	19.9 (9.4)	22.6 (8.5)		23.2 (8.8)	24.3 (9.2)
**W3T1**	15.8 (8.1)	18.1 (8.5)	21.5 (7.6)	22.0 (9.2)		24.8 (9.3)
**W3T2**	15.6 (7.6)	16.3 (7.9)	18.6 (7.6)	22.8 (8.9)	22.5 (8.4)	

Table S7. Trial-level subject identification rates for development-evaluation intervals.
An average trial-level subject identification rate (%) is presented for each development-evaluation interval. Standard deviations are in parentheses.

	**30 M**	**1 W**	**2 W**
**Free**	19.8 (8.6)	19.5 (8.8)	14.4 (7.3)
**Task**	23.0 (8.4)	20.5 (8.9)	17.0 (8.2)

Table S8. Trial-level subject identification rates with and without time warping.

Average trial-level subject identification rates (%) with time warping (DTW) and without time warping (Euclidean) are presented. Standard deviations are in parentheses.

		30 M	1 W	2 W
**Free**	**DTW**	19.8 (8.6)	19.5 (8.8)	14.4 (7.3)
	**Euclidean**	16.9 (8.2)	16.9 (7.9)	13.0 (7.0)
**Task**	**DTW**	23.0 (8.4)	20.5 (8.9)	17.0 (8.2)
	**Euclidean**	19.6 (8.2)	17.9 (9.0)	14.7 (7.4)

Table S9. Session-level subject identification rates (%) for each development-evaluation composition.

Free

Dev. \ Eval.	W1F1	W1F2	W2F1	W2F2	W3F1	W3F2
**W1F1**		77.8	81.5	66.7	63.0	44.4
**W1F2**	81.5		77.8	63.0	63.0	66.7
**W2F1**	66.7	70.4		77.8	77.8	66.7
**W2F2**	74.1	70.4	74.1		81.5	81.5
**W3F1**	63.0	66.7	88.9	88.9		74.1
**W3F2**	51.9	55.6	66.7	77.8	74.1	

Task

Dev. \ Eval.	W1T1	W1T2	W2T1	W2T2	W3T1	W3T2
**W1T1**		92.6	77.8	70.4	81.5	59.3
**W1T2**	74.1		77.8	70.4	70.4	70.4
**W2T1**	74.1	74.1		92.6	85.2	77.8
**W2T2**	55.6	77.8	88.9		81.5	81.5
**W3T1**	66.7	70.4	85.2	81.5		81.5
**W3T2**	55.6	55.6	70.4	81.5	77.8	

Table S10. Session-level subject identification rates for development-evaluation intervals.
Session-level subject identification rates (%) are averaged and presented for each development-evaluation interval. Standard deviations are in parentheses.

	30 M	1 W	2 W
**Free**	76.5 (3.0)	75.0 (8.2)	59.3 (7.9)
**Task**	84.6 (7.9)	76.4 (7.4)	66.2 (8.9)

Table S11. Comparisons of correct identification decisions between trial groups.

Average ratios of correct subject identification decisions are compared between the trial groups. One-sided unpaired *t*-test with unequal sample sizes and unequal variances and Cohen's *d* with unequal sample sizes and unequal variances are presented with respect to development-evaluation intervals and session conditions.

Free

	t	*d.f.*	*P*	*Cohen's d*
Between self-face and other-face				
**30 M**	0.69	190.1	0.245	0.08
**1 W**	3.22	515.9	0.001	0.23
**2 W**	2.53	238.2	0.006	0.26
Between self-face and no-face				
**30 M**	1.00	201.6	0.158	0.12
**1 W**	3.09	546.0	0.001	0.22
**2 W**	2.28	236.6	0.012	0.23
Between other-face and no-face				
**30 M**	0.61	317.2	0.273	0.07
**1 W**	-0.14	849.2	0.556	-0.01
**2 W**	-0.58	429.9	0.720	-0.06

Task

	t	*d.f.*	*P*	*Cohen's d*
Between self-face and other-face				
**30 M**	1.73	180.5	0.043	0.20
**1 W**	3.85	479.9	<0.001	0.28
**2 W**	2.75	271.3	0.003	0.28
Between self-face and no-face				
**30 M**	2.60	187.1	0.005	0.31
**1 W**	4.29	499.1	<0.001	0.31
**2 W**	3.53	245.0	<0.001	0.36
Between other-face and no-face				
**30 M**	1.83	317.9	0.034	0.20
**1 W**	1.00	851.4	0.158	0.07
**2 W**	1.32	407.4	0.093	0.13

Table S12. Comparisons of correct identification decisions between time intervals for each trial group.

The average ratios of correct subject identification decisions are compared between development-evaluation time intervals for each trial group, separately. One-sided unpaired *t*-test with unequal sample sizes and unequal variances and Cohen's *d* with unequal sample sizes and unequal variances are presented with respect to the trial groups and session conditions.

Free

	t	*d.f.*	*P*	*Cohen's d*
**Self-face trials**				
Between 30 M and 1 W	-0.97	289.7	0.835	-0.09
Between 1 W and 2 W	1.85	400.0	0.032	0.16
Between 30 M and 2 W	0.70	327.2	0.242	0.08
**Other-face trials**				
Between 30 M and 1 W	0.88	310.1	0.190	0.08
Between 1 W and 2 W	5.72	489.7	<0.001	0.45
Between 30 M and 2 W	5.46	333.3	<0.001	0.57
**No-face trials**				
Between 30 M and 1 W	0.00	310.1	0.500	<0.01
Between 1 W and 2 W	5.00	550.2	<0.001	0.38
Between 30 M and 2 W	3.94	303.1	<0.001	0.42

Task

	t	*d.f.*	*P*	*Cohen's d*
**Self-face trials**				
Between 30 M and 1 W	0.58	265.7	0.281	0.06
Between 1 W and 2 W	1.16	432.1	0.124	0.10
Between 30 M and 2 W	1.45	301.3	0.074	0.16
**Other-face trials**				
Between 30 M and 1 W	3.41	285.1	<0.001	0.32
Between 1 W and 2 W	2.55	383.1	0.006	0.22
Between 30 M and 2 W	4.95	364.2	<0.001	0.51
**No-face trials**				
Between 30 M and 1 W	1.72	291.5	0.044	0.16
Between 1 W and 2 W	3.60	508.2	<0.001	0.28
Between 30 M and 2 W	4.41	307.5	<0.001	0.47

Supplementary text

Within-subject scanpath comparisons

Each of the 27 subjects observed an image set six times in Free and six times in Task sessions throughout our experiment, resulting in 15 possible non-redundant within-subject scanpath pairs for each session condition. Thus, there were overall 405 within-subject scanpath pairs from every subject for each image set and each of Free and Task conditions. However, dissimilarity values from comparisons between valid eye movements were considered in the analysis.

Between-subject scanpath comparisons

In total, there were 12,636 non-redundant between-subject scanpath pairs for each image set and each of the Free and Task conditions. Sessions with the same session names (e.g., W1F1 session of subject #1 and that of subject #2) were also paired and analysed as pairs with 30 M interval. The distributions of dissimilarity values from between-subject scanpath pairs were plotted and compared with the distributions from within-subject pairs (Figs. 4, a-d, yellow versus purple plots). The scanpath dissimilarities of within-subject pairs were smaller than those of between-subject pairs. Distribution differences were measured with KL divergence (Supplementary Table S2).

Subject distinguishability of image sets according to their image compositions

To investigate the effects of image set compositions on scanpath consistency, we defined and analysed the subject distinguishability of image sets. The ratio of the overall average between-subject scanpath dissimilarity to the overall average within-subject scanpath dissimilarity value was defined as the subject distinguishability of each image set. The image sets used in our experiment were divided based on different criteria (Supplementary Fig. S2-4). Among these criteria, only one criterion exhibited a significant effect on distinguishability, which was the presence of human face images. Image sets including more than one human face evoked more robust idiosyncratic scanpaths than those without a human face. However, distinguishability was not dependent on the number of faces in an image set.

Trial groups with self-faces

Trials from each session were divided into the “self-face,” “other-face,” and “no-face” groups. The self-face group included three trials for each session for each subject, with each image set containing one of the subject’s self-face images, which was solicited before the experiment. The other-face group included 57 trials with each containing at least one human (non-self) face image. In addition, the no-face group included 50 trials with no human face images. The trials in the self-face and other-face groups were different for each subject and those in the no-face groups were identical for every subject. Dissimilarity values from within-subject scanpath comparisons were divided into these three trial groups (self-face, other-face, and no-face), and their distributions were plotted (Figs. 4, e-h). The distributions of the self-face group were different from those of the other-face and no-face groups as confirmed with KL divergence (Supplementary Table S4).

Subject identification method

The scanpaths of evaluation sessions (evaluation scanpaths) were compared with the corresponding scanpaths of development sessions (development scanpaths). Subjects were identified during trial-level subject identification using the nearest neighbouring method[47], i.e., evaluation scanpaths were matched with the corresponding identities of minimally dissimilar development scanpaths.

To confirm distinctiveness in each subject's preferences during experiment, we first conducted subject identification with their preferences. A single preference answer cannot represent an identity because there were only four possible choices for each selection; therefore, a sequence of preferred image indices of each session was compared to the others, and an evaluation sequence followed the identity of the closest development sequence. On average, identification performance was 88.3% with 30 M interval between evaluation and development data.

Dissimilarity between a development scanpath with identity ID_j and an evaluation scanpath was defined as s_j, where $j = 1,2, \ldots, N_{sub}$ and N_{sub} is the number of subjects ($N_{\mathrm{sub}} = 27$). A decision d_J, which identifies a scanpath as identity ID_J or not, can be expressed as

$$d_J = \begin{cases} 1\,, & if\;\; s_J = \min_j s_j \\ 0\,, & otherwise \end{cases} .$$

If an evaluation scanpath shares the minimum dissimilarity with the development scanpath with identity ID_J, it is identified as ID_J, and d_J is 1 (there was no case with multiple minimum dissimilarities). When an evaluation session was tested, each valid scanpath (maximum 110 in total) was identified. In a development-evaluation composition, 27 evaluation sessions (one from each subject) were tested with another 27 sessions from development data. In this way, a trial identification rate can be determined for each image index, telling us how many subjects (up to 27) were correctly identified with the valid scanpaths induced by that image set. These rates can then be compared against chances, with each chance level of identification rate equivalent to one over the number of candidate identities for each identification (corresponding to valid development scanpaths and varying due to the number of valid scanpaths for each trial index, minimum chance level=1/27).

The results from trial-level subject identification were gathered for session-level subject identification. A decision was made by a majority vote[48] on candidate identities for each session, with each identification decision for a valid scanpath voting for a candidate identity. The identification decision for a scanpath with index t of a session can be construed as $d_{t,j} \in \{0, 1\}, t = 1, 2, \ldots, N_{\mathrm{tr}}$, and $j = 1, 2, \ldots, N_{\mathrm{sub}}$, where N_{tr} is the number of trials ($N_{\mathrm{tr}} = 110$). When a scanpath with index t is identified as identity ID_j, $d_{t,j}$ is 1, otherwise 0. For an invalid scanpath, $d_{t,j} = 0$ for every j. Thus, an identity was assigned as follows for every evaluation session: ID_J is selected if

$$J = \underset{j}{\operatorname{argmax}} \sum_{t=1}^{N_{\mathrm{tr}}} d_{t,j} .$$

Each identity decision for a valid scanpath voted on an identity among N_{sub} candidate identities, and identity ID_J was selected according to the maximum votes. When multiple ID_Js gained the same maximum number of votes, the final identity ID_{J^*} was determined by comparing dissimilarities.

$$J^* = \underset{J}{\operatorname{argmin}} \sum_{t=1}^{N_{\mathrm{tr}}} d_{t,J} s_{t,J} \ ,$$

where $s_{t,J}$ is the dissimilarity value between the scanpath with index t of an evaluation session and that of a development session with ID_J.

<u>Subject identification with extended development data</u>

Identification decisions are not reliable when an evaluation scanpath is tested against a single scanpath from each subject. Therefore, we also checked idiosyncrasies in eye movements using larger development sets for subject identification to assess the potential of our method in more realistic human identification conditions. We built development data sets consisting of five scanpaths for each image set from each subject. Then, the remaining scanpath for each image set for each subject was used as evaluation data, e.g., an evaluation scanpath from session W1T1 was compared against all valid scanpaths from every other session, namely, W1T2, W2T1, W2T2, W3T1, and W3T2. In this way, each evaluation scanpath was tested against (up to) 135 development scanpaths rather than only (up to) 27 in the initial analysis (depending on the number of valid scanpaths for each image set and identity). This approach thus serves as a six-fold validation test utilizing five times more data than those in the original analysis. In total, there were six development-evaluation compositions for each of Free and Task sessions (Supplementary Fig. S6A). Each evaluation scanpath was compared with the corresponding scanpaths in development data and identified with its nearest neighbouring scanpath. Trial-level identification decisions were compiled, and session-level identification decisions were rendered by a majority vote as described in the preceding section (Supplementary Fig. S6B).

<u>Additional data acquisition and scanpath comparisons with a longer time interval</u>

To observe consistency of individual scanpaths over a longer period, we conducted the same experiment one year after the original experiment with the same subjects. Fourteen of the 27 original subjects participated this additional experiment. The experimental settings, paradigm, and image sets were the same as the original experiment, but only Task sessions were recorded. Up to six sessions were recorded for each subject (a subject could conduct two, another could conduct four, the other two could conduct five, and the rest conducted six sessions); overall, 76 additional Task sessions were obtained. Scanpaths of the additional Task sessions were compared with those of the original Task sessions, and their interval was defined as “one year” (1 Y). We observed the dissimilarity value distributions for scanpath pairs with 1 Y interval (Fig. 6).

During the subject identification for 1 Y interval between development and evaluation data, the additional data were identified with the original data as development. All of the 27 subjects’ data in the original data were used; thus, the minimum chance

level was also 1/27 for these development-evaluation compositions. In this analysis, the number of development sessions per subject varied from one to six. When k development sessions per subject were used, there were ${}_6\mathrm{C}_k$ subsets where k sessions were chosen among six original sessions (W1T1, W1T2, W2T1, W2T2, W3T1, and W3T2). Overall, average trial-level subject identification rates according to the development set sizes were demonstrated (Supplementary Fig. S7). With multiple development sessions per subject, trials in each evaluation session were identified as the method in the Subject Identification with Extended Development Data section.

References

1. Weichselgartner, E. & Sperling, G. Dynamics of automatic and controlled visual attention. *Science* **238**, 778-780 (1987).
2. Kowler, E., Anderson, E., Dosher, B. & Blaser, E. The role of attention in the programming of saccades. *Vision Res.* **35**, 1897-1916 (1995).
3. Peterson, M., Kramer, A. & Irwin, D. Covert shifts of attention precede involuntary eye movements. *Percept. Psychophys.* **66**, 398-405 (2004).
4. Theeuwes, J. & Belopolsky, A. V. Reward grabs the eye: oculomotor capture by rewarding stimuli. *Vision Res.* **74**, 80-85 (2012).
5. Yarbus, A. L. Eye movements during perception of complex objects in *Eye Movements and Vision* 171-211 (Plenum Press, 1967).
6. Borji, A. & Itti, L. Defending Yarbus: eye movements reveal observers' task. *J. Vision* **14**, 29 (2014).
7. Sugano, Y., Ozaki, Y., Kasai, H., Ogaki, K. & Sato, Y. Image preference estimation with a data-driven approach: a comparative study between gaze and image features. *Eye Movement Res.* **7**, 1-9 (2014).
8. Zangemeister, W. H., Sherman, K. & Stark, L. Evidence for a global scanpath strategy in viewing abstract compared with realistic images. *Neuropsychologia* **33**, 1009-1025 (1995).
9. Field, M. & Cox, W. M. Attentional bias in addictive behaviors: a review of its development, causes, and consequences. *Drug Alcohol Depend.* **97**, 1-20 (2008).
10. Castellanos, E. H. *et al*. Obese adults have visual attention bias for food cue images: evidence for altered reward system function. *Int. J. Obes.* **33**, 1063-1073 (2009).
11. Mogg, K., Bradley, B. P., Field, M. & De Houwer, J. Eye movements to smoking-related pictures in smokers: relationship between attentional biases and implicit and explicit measures of stimulus valence. *Addiction* **98**, 825-836 (2003).
12. Noton, D. & Stark, L. Scanpaths in eye movements during pattern perception. *Science* **171**, 308-311 (1971).
13. Josephson, S. & Holmes, M. E. Visual attention to repeated internet images: testing the scanpath theory on the world wide web. *Proc. ETRA 2002*, 43-49 (2002).
14. Itti, L. & Koch, C. Computational modelling of visual attention. *Nat. Rev. Neurosci.* **2**, 194-203 (2001).
15. Cantoni, V., Faldi, C., Nappi, M., Porta, M. & Ricco, D. GANT: gaze analysis technique for human identification. *Pattern Recogn.* **48**, 1027-1038 (2015).
16. Rigas, I. & Komogortsev, O. Biometric Recognition via Probabilistic Spatial Projection of Eye Movement Trajectories in Dynamic Visual Environments. *IEEE Trans. Inf. Forensics Security* **9**, 1743-1754 (2014).
17. Pieters, R. & Warlop, L. Visual attention during brand choice: the impact of time pressure and task motivation. *Int. J. Res. Mark.* **16**, 1-16 (1999).

18. Ji, Q., Zhu, Z. & Lan, P. Real-time nonintrusive monitoring and prediction of driver fatigue. *IEEE Trans. Veh. Technol.* **53**, 1052-1068 (2004).

19. Judd, T., Durand, F. & Torralba, A. Fixations on low-resolution images. *J. Vision* **11**, 14 (2011).

20. Walker-Smith, G., Gale, A. & Findlay, J. Eye movement strategies involved in face perception. *Perception* **6**, 313-326 (1977).

21. Park, J., Shimojo, E. & Shimojo, S. Roles of familiarity and novelty in visual preference judgments are segregated across object categories. *Proc. Natl. Acad. Sci. U.S.A.* **107**, 14552-14555 (2010).

22. Shimojo, S., Simion, C., Shimojo, E. & Scheier, C. Gaze bias both reflects and influences preference. *Nat. Neurosci.* **6**, 1317-1322 (2003).

23. Mitsuda, T. & Glaholt, M. G. Gaze bias during visual preference judgements: effects of stimulus category and decision instructions. *Vis. Cogn.* **22**, 11-29 (2014).

24. Glaholt, M. G. & Reingold, E. M. Eye movement monitoring as a process tracing methodology in decision making research. *J. Neurosci. Psychol. Econ.* **4**, 125-146. (2011).

25. Wolford, G. & Morrison, F. Processing of unattended visual information. *Mem. Cognition* **8**, 521-527 (1980).

26. Devue, C. & Brédart, S. Attention to self-referential stimuli: can I ignore my own face? *Acta Psychol.* **128**, 290-297 (2008).

27. Althoff, R. R. & Cohen, N. J. Eye-movement-based memory effect: a reprocessing effect in face perception. *J. Exp. Psychol.-Learn. Mem. Cogn.* **25**, 997-1010 (1999).

28. Barton, J. J., Radcliffe, N., Cherkasova, M. V., Edelman, J. & Intriligator, J. M. Information processing during face recognition: the effects of familiarity, inversion, and morphing on scanning fixations. *Perception* **35**, 1089-1105 (2006).

29. Phillips, P. J., Moon, H., Rizvi, S. A. & Rauss, P. J. The FERET evaluation methodology for face-recognition algorithms. *IEEE Trans. Pattern Anal. Mach. Intell.* **22**, 1090-1104 (2000).

30. Russakovsky, O. *et al.* Imagenet large scale visual recognition challenge. *Int. J. Computer Vision* **115**, 211-252 (2015).

31. Winkler, S. & Ramanathan, S. Overview of eye tracking datasets. *Proc. QoMEX 2013*, 212-217 (2013).

32. Duchowski, A. Visual psycolphysics in *Eye Tracking Methodology: Theory and Practice (2nd ed.)* 29-39 (Springer Science & Business Media, 2007).

33. Tobii Technology AB, User manual–Tobii Studio, Manual Ver. 3.2, Rev A. 11 (2012).

34. Anderson, N. C., Anderson, F., Kingston, A. & Bischof, W. F. A comparison of scanpath comparison methods. *Behav. Res. Meth.* **47**, 1377-1392 (2015).

35. Pieters, R., Rosbergen, E. & Wedel, M. Visual attention to repeated print advertising: a test of scanpath theory. *J. Marketing Res.* **36**, 424-438 (1999).

36. Sakoe, H. & Chiba, S. Dynamic programming algorithm optimization for spoken word recognition. *IEEE Trans. Acoust., Speech, Signal Process.* **26**, 43-49 (1978).

37. Turetsky, R. J. & Ellis, D. P. Ground-truth transcriptions of real music from force-aligned midi syntheses. *Proc. ISMIR 2003,* 135-141 (2003).

38. Levenshtein, V. I. Binary codes capable of correcting deletions, insertions, and reversals. *Soviet Physics Doklady* **10**, 707-710 (1966).

39. Brandt, S. A. & Lawrence, W. S. Spontaneous eye movements during visual imagery reflect the content of the visual scene. *J. Cognitive Neurosci.* **9**, 27-38 (1997).

40. Cristino, F., Mathôt, S. Theeuwes, J. & Gilchrist, I. D. ScanMatch: a novel method for comparing fixation sequences. *Behav. Res. Meth.* **42**, 692-700 (2010).

41. Ellis, D. Dynamic Time Warp (DTW) in Matlab. *Columbia University* http://www.ee.columbia.edu/~dpwe/resources/matlab/dtw/ (2003).

42. Cohen, J. *Statistical power analysis for the behavioural sciences (Rev. ed.)* (Academic Press Inc., 1977).

43. Kullback, S. *Information Theory and Statistics,* (Dover Publications Inc., 1968).

44. Little, A. C., Jones, B. C. & DeBruine, L. M. Facial attractiveness: evolutionary based research. *Phil. Trans. R. Soc. B* **366**, 1638-1659. (2011).

45. Fuhrmann, D. *et al*. Perception and recognition of faces in adolescence. *Sci. Rep*. **6**, 33497; 10.1038/srep33497 (2016).

46. Jain, A. K., Ross, A. & Prabhakar, S. An introduction to biometric recognition. *IEEE Trans. Circuits, Syst. Video Technol.* **14**, 4-20 (2004).

47. Cover, T. M. & Hart, P. E. Nearest neighbor pattern classification. *IEEE Trans. Inf. Theory* **13**, 21-27 (1967).

48. Polikar, R. Ensemble based systems in decision making. *IEEE Circuits Syst. Mag.* **6**, 21-45 (2006).
